# Efficacy and Effectiveness of High Molecular Weight Non‐Cross‐Linked Hyaluronic Acid Plus Succinic Acid Mesotherapy in Rosacea as Adjunct Therapy

**DOI:** 10.1111/jocd.70484

**Published:** 2025-10-02

**Authors:** Alberto Leguina‐Ruzzi, Alejandro Navarro, Marjorie Zambrano

**Affiliations:** ^1^ Faculty of Medicine, Universidad Andrés Bello Santiago Región Metropolitana Chile; ^2^ TotalSkin Clinic Santiago Chile; ^3^ Faculty of Medical Sciences, Universidad de Guayaquil, Escuela de Medicina Guayaquil Ecuador

**Keywords:** adjunct therapy, dermatosis, high molecular weight hyaluronic acid, mesotherapy, rosacea, skin quality, succinic acid

## Abstract

**Background:**

Rosacea is a highly prevalent dermatosis affecting 5% of the world's population. Its impact is not only physical but also psychological, as over 60% of patients with rosacea also suffer from depression, anxiety, and other psychological comorbidities. Currently, topical treatments yield low or unsatisfactory success rates, and rosacea complications such as disfigurement, burning sensation, and pain can greatly affect patients' well‐being. Intra‐dermotherapy with high molecular weight non‐cross‐linked hyaluronic acid plus succinic acid has shown anti‐inflammatory, regenerative, and vascular modulatory effects.

**Objectives:**

To evaluate the efficacy and effectiveness of high molecular weight non‐cross‐linked hyaluronic acid (HMWNCHA) plus succinic acid (SA) mesotherapy in rosacea patients who are not responding to topical treatments. To determine the degree of improvement in general parameters such as erythema, telangiectasia, and skin laxity and hydration. To compare the cost of this treatment with that of laser therapy, a widely used modality.

**Methods:**

We performed a non‐randomized, controlled interventional study, and we evaluated the results after three sessions (once monthly) as the primary endpoint of this mesotherapy, using a product containing 1.1% of high molecular weight non‐cross‐linked hyaluronic acid plus 1.6% succinic acid (full face, 2 mL per session, administered using the papule technique) in a group of 20 male and female patients, aged 40–45 years, Fitzpatrick skin types I–III, without comorbidities. All patients were already undergoing topical treatment with Ivermectin 1%, Metronidazole 0.75%, and photoprotection, but without good results or clear improvement, quantified as a reduction in the Investigator's Global Assessment (IGA) severity score of at least 1 point. Erythema, hydration, and facial telangiectasia were evaluated at every session for proper quantification. Our study group was compared with a control group that only continued their topical treatment, and the study lasted 4 months, with a prospective follow‐up at 6 months.

**Results:**

Our results showed a reduction of 80% in the baseline erythema, an improvement of 30% in skin hydration, and a reduction of 20% in facial telangiectasia after three sessions, compared with patients who used only topical treatment. In addition, the costs were significantly lower than those of three sessions of laser therapy in a private healthcare setting.

**Conclusion:**

We demonstrated that using this acid combination in mesotherapy is an efficient, safe, and cost‐effective adjunct therapy for patients already using a particular topical treatment.

## 1 Introduction

Rosacea is a chronic inflammatory dermatosis characterized by skin lesions such as erythema and papulopustular elements, phymas, as well as ocular lesions [1]. This is an angioneurosis localized mainly in the centrofacial innervation zone of the trigeminal nerve and is caused by various factors that can be grouped as follows: vascular disorders, changes in the dermal connective tissue, microorganisms, digestive tract dysfunctions, immune disorders, changes in the pilosebaceous system, oxidative stress, climatic factors, and psychovegetative disorders [2].

It is well established that several exogenous triggers can exacerbate rosacea, such as dietary (alcohol, hot, spicy foods), solar radiation, wind, extreme temperatures, intense physical activity, emotional stress, certain cosmetics, and prolonged use of steroid ointments, among others [3]. However, there are also endogenous factors that may contribute to these clinical manifestations, such as Demodex folliculorum mites and gastrointestinal, endocrine, nervous system, and immunological disorders [4].

The worldwide prevalence of rosacea has been estimated to range from 2.3% to 22%, affecting mostly women between 25 and 65 years of age, with Fitzpatrick phototypes I and II, particularly in northeastern Europe [5, 6].

The complex molecular mechanism underlying rosacea has been established and it involves neurovascular and innate immune dysregulation. Exposomes and genetic predispositions activate a cascade of pro‐inflammatory cytokines, growth factors, and overactivation of transient receptor potential (TRPV) cation channels, leading to the main clinical manifestations: erythema, flushing, tissue hypertrophy, papules and pustules, sensory changes (soreness, burning, itching, tingling), and dryness [7, 8, 9].

The emotional and psychosocial impact has been well‐established as a key burden of rosacea. These patients tend to experience significantly diminished quality of life and higher rates of anxiety and depression compared to individuals unaffected by the condition [10, 11].

The international clinical guidelines for rosacea patient management (ROSCO treatment algorithm) are considered among the most trustworthy guidelines followed by medical doctors [12]. However, topical treatment provides a relatively low or unsatisfactory success rate with a high likelihood of relapsing episodes [13]. For that reason, adjunctive therapies have emerged as an important part of rosacea management, with laser therapy leading in effectiveness [14]; unfortunately, due to its high costs, access remains limited for most patients.

Intradermal therapy, also known as mesotherapy, is a technique used to inject drugs into the superficial layer of the skin. It has been widely used for the past 40 years for multiple purposes, including pain management, antibiotic administration, antiaging, and depigmentation, among other applications [15]. In rosacea, botulinum toxin mesotherapy has been shown to reduce flushing [16] and low molecular weight cross‐linked hyaluronic acid has been shown to reduce erythema [17].

High molecular weight non‐cross‐linked hyaluronic acid (HMWNCHA) has demonstrated anti‐inflammatory, pro‐regenerative, and bacteriostatic effects [18, 19, 20, 21]; thus, it is the main ingredient in multiple antiaging mesotherapy formulations [22]. On the other hand, succinic acid (SA), due to its metabotropic and energotropic properties, has been proposed to act via the hypoxic salvage pathway [23]. The combination of HMWNCHA and SA as mesotherapy is currently used as a skin rejuvenation approach with promising results [24, 25]. In 2020, a preclinical study in Ukraine, including three patients with rosacea who were treated by the Hyalual company, showed an improvement in appearance after three sessions; however, no in‐depth evaluations were performed, nor were the cases published. Despite this pilot approach, no further studies have been conducted in the field of rosacea.

The aim of our study was to evaluate the efficacy and effectiveness of high molecular weight non‐cross‐linked hyaluronic acid plus succinic acid mesotherapy in rosacea patients who are not responding to topical treatments, to determine the degree of improvement in general parameters such as erythema, telangiectasia, and skin laxity and hydration, and to compare the cost of this treatment with that of laser therapy, a commonly used therapy.

## 2 Materials and Methods

### 2.1 Study Population and Design

In a non‐randomized controlled interventional setting study, 40 male and female patients suffering from rosacea were studied in two groups. The study lasted 4 months, with a prospective follow‐up at 6 months. Their demographic and clinical characteristics are summarized in Table 1.

**TABLE 1:** Clinical and demographic characteristics of the study population.

	Control group (*n* = 20)	Intervention group (*n* = 20)	*p*
Age (years)	41.2 ± 1.2	40.5 ± 1.5	0.87
Gender	Male = 8	Male = 6	n/a
	Female = 12	Female = 14	
IGA grade	3.2 ± 0.2	3.1 ± 0.1	0.89
Fitzpatrick scale	1 = 8	1 = 10	n/a
	2 = 6	2 = 6	
	3 = 6	3 = 4	

*Note:* A total of 40 patients with moderate rosacea were enrolled. There were no statistically significant differences between groups in age, sex distribution, IGA severity score, or Fitzpatrick skin type, confirming baseline comparability. All data are presented as mean ± standard error of the mean except where indicated.

All patients were treated for 1 month with Ivermectin 1% cream at night, Metronidazole 0.75% gel during the day, and photoprotection (FotoUltra Redness SPF50 ISDIN). All participants were advised on lifestyle modifications and behavioral strategies to manage symptoms and minimize triggers. These strategies included stress management techniques, gentle facial massage, and avoiding known triggers like extreme temperatures and spicy foods.

All patients were reevaluated 1 month after. Those who experienced subjective unsatisfactory results and showed no clinical signs of improvement, quantified as no reduction of the Investigator's Global Assessment (IGA) severity score of at least 1 point, were offered an experimental mesotherapy treatment while continuing with their topical regimen using the gold standard treatment. Twenty patients agreed to initiate this adjunct treatment and were included in the ‘intervention group’. The remainder, who did not agree to injectable treatment and did not seek other intervention (IPL, laser, oral isotretinoin, or other medications), continued with the same topical treatment and were followed up monthly as the ‘control group’.

Our study excluded participants with chronic diseases such as diabetes, obesity, hypertension, autoimmune disorders, coagulopathies, or intercurrent inflammatory or infectious disorders. Smokers and users of recreational or non‐recreational drugs (mainly statins, antibiotics, isotretinoin, and anti‐inflammatory drugs) were also eliminated. Patients suffering from other skin disorders such as acne, psoriasis, or dermatitis were excluded as well. Patients who were pregnant, trying to become pregnant, or breastfeeding were also excluded. Individuals with known allergies to anesthetics or components of the mesotherapy product were also excluded from the study (Table 2).

**TABLE 2:** Exclusion criteria.

Exclusion criteria
Diabetes
Obesity
Hypertension
Autoinmmune disorders
Coagulopathies
Intercurrent inflammatory or infectious disease
Smokers
Users of recreational or non‐recreational drugs (statins, antibiotics, isotretinoin, and anti‐inflammatory drugs)
Acne
Psoriasis
Dermatitis
Pregnancy
Breastfeeding
Allergies to anesthetics
Allergies to any of the components of the mesotherapy product

All patients were scored using the IGA severity score [26]. Most of them were categorized as having mild to moderate rosacea. Patients presenting severe rosacea were treated according to ROSCO guidelines and were excluded from this study.

Each subject signed a comprehensive written informed consent form, which included permission to obtain and publish their photographs. This work was conducted in accordance with the principles outlined in the Declaration of Helsinki.

The study was set in a private practice setting and an internal board of consultant physicians approved and supported the study under the code RCM56742, year 2021.

### 2.2 Injection Technique: HMWNCHA Plus SA Mesotherapy

A group of 20 patients was treated with three sessions of HMWNCHA plus SA mesotherapy as the primary interventional endpoint (based on the Hyalual company's pre‐established application protocols). Secondary endpoints included the IGA severity score and associated clinical manifestations. The sessions were performed at one‐month intervals.

Before the injections, patients' skin was disinfected using 2% chlorhexidine, followed by the application of a topical anesthetic cream (lidocaine 2%, prilocaine 7%, and tetracaine 6%) for 30 min under occlusion. The skin was then cleansed again using 2% chlorhexidine. The mesotherapy product used was Xela Rederm 1.1% (Hyalual, Diaco Biopharmaceutical, Italy), supplied as 2 mL prefilled syringes containing HMWNCHA 11 mg/mL plus 16 mg/mL succinic acid. One full syringe was used for the entire face, employing a micro‐papule technique using the 30G 13 mm needles included in the product. After the procedure, a gentle massage was performed using the product ProfiDelux (Hyalual, Diaco Biopharmaceutical, Italy), a sterile water‐based spray containing hyaluronic and succinic acid. Patients were advised to avoid sun exposure and to resume their regular topical treatment 12 h after the mesotherapy session. Patients continued using Ivermectin 1% cream at night, Metronidazole 0.75% gel during the day, and photoprotection 24 h post mesotherapy application.

### 2.3 Adverse Event Reporting

The side effects and complications were recorded during the study time. The incidence rate was calculated as reported cases divided by the total person‐time at risk during a specific period (number 60 as 3 sessions of treatments were performed in 20 patients) multiplied by a population coefficient of 100.

### 2.4 Photo Documentation

High‐resolution digital photographs were taken and collected using an iPhone 14 Pro Max camera, maintaining angle, distance, background, and lighting as consistent as possible.

### 2.5 Measurement of Erythema, Telangiectasia, Hydration, and Skin Elasticity

For erythema and telangiectasia, the photographs were analyzed using the ImageJ software (https://imagej.net/ij/).

The samples for skin hydration assessments were collected using a multiple‐probe device for skin measurements (Courage‐Khazaka Electronic GmbH, Germany), under standardized conditions of temperature and humidity (20°C–22°C and 40%–60%, respectively), after a rest period of 15–20 min for each subject. The hydration level was determined by the capacitance method using the Corneometer CM 825, following a previously standardized protocol [27].

Skin elasticity was measured using an indentometer device (ElastiMeter by Delfin, Kuopio, Finland). This parameter was measured four times for each individual, with the mean value used as the final result. Environmental conditions were room temperature 22°C–25°C and humidity 30%–50% [28].

All measurements were normalized to percentage relative to time 0 (before treatment).

### 2.6 Sensitive Symptoms Assessment

Itchiness, soreness, burning, and tingling were quantified using self‐perception numerical rating scales (NRS). During each visit, patients were asked to assess their symptoms on a validated 0 to 10 scale previously published, validated, and widely used for evaluating multiple pathologies [29, 30, 31, 32].

### 2.7 Ex Vivo Confocal Scanning Laser Microscopy

Samples were visualized using a Leica LPE confocal laser microscope with a previously established ex vivo technique [33].

A pre‐treatment skin sample from one consenting patient was collected using a 3 mm biopsy punch. The area selected was actively affected by rosacea and was aesthetically acceptable to avoid scarring. A second sample was taken 1 month after the third mesotherapy session, from an area adjacent to the original site. Sample processing and evaluation were performed in the Department of Mitochondrial Physiology, No. 75, Institute of Physiology of the Czech Academy of Sciences, former affiliation of the corresponding author.

### 2.8 Cost Comparisons

Ten randomly selected clinics in Santiago, Chile, were contacted by phone to inquire about the price per session with laser/IPL or mesotherapy treatment for rosacea using the commercially available product registered by the Instituto de Salud Publica (ISP), Xela Rederm 1.1%. Costs were converted from the local currency to Euros at the exchange rate current at the time of writing of this paper. The graph shows mean value and standardized mean difference (SM).

### 2.9 Statistical Analysis

The data were analyzed with Prism v.10.4.2 (GraphPad software) and are presented as mean–standard error of the mean. Differences between the means of two groups were analyzed using the nonparametric Kruskal–Wallis or Wilcoxon tests, corrected by Tukey's HSD method. Normality was checked using the Shapiro–Wilk test, and sample size was calculated using the free software OpenEpi v3.01 (Kevin M. Sullivan, Emory University based on John C. Pezzullo code, 2013). A *p* value < 0.05 was defined as statistically significant.

## 3 Results

### 3.1 Erythema, Telangiectasia, Hydration, and Skin Elasticity

**FIGURE 1:**
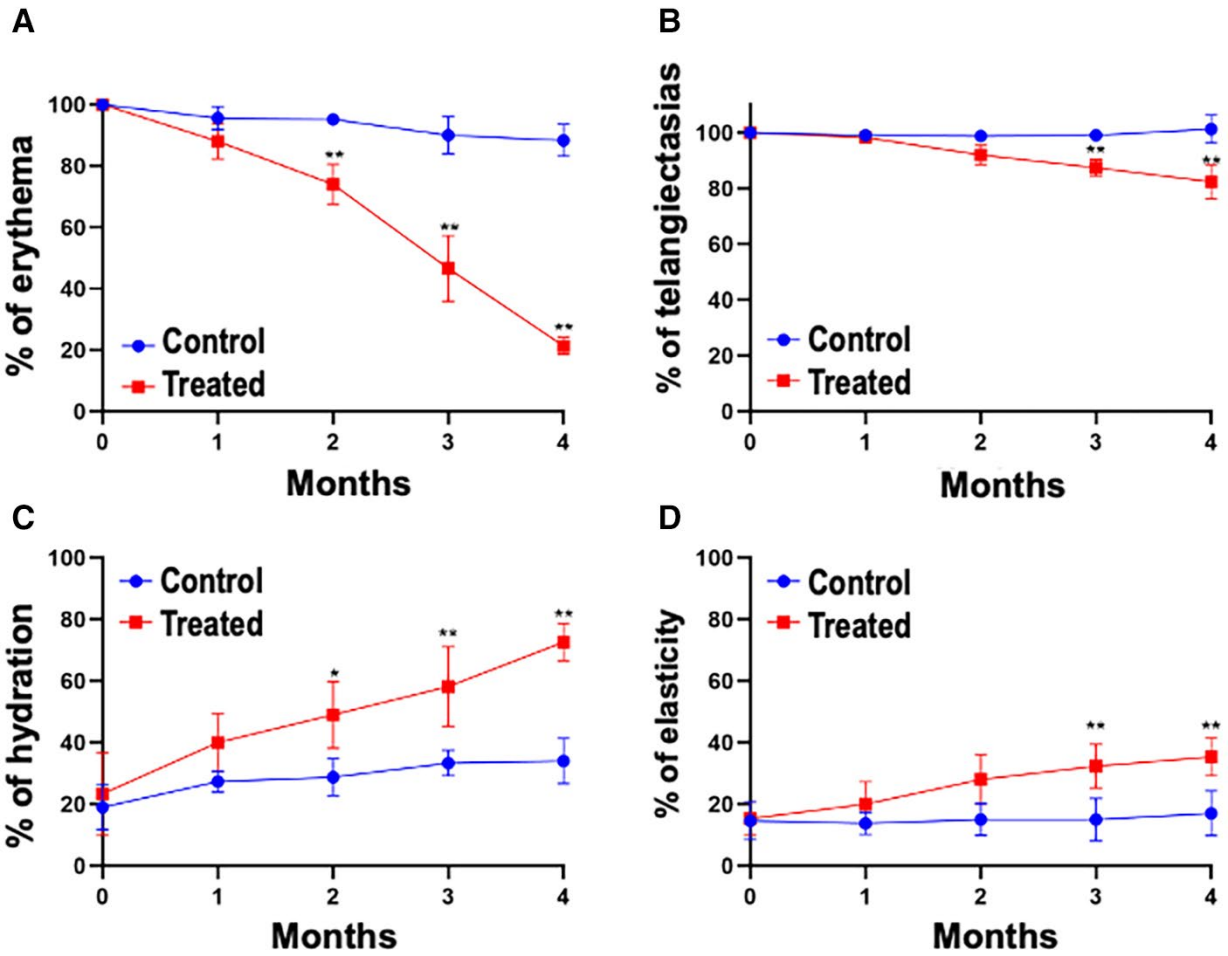
Percentages for (A) erythema, (B) telangiectasias, (C) skin hydration, and (D) elasticity measured at baseline and monthly for 4 months. The treated group showed statistically significant improvement in all parameters compared to the control group. Data are presented as mean ± standard error of the mean Kruskal–Wallis test *p < 0.05, **p < 0.01.

Participants treated with the mesotherapy protocol showed a significant and progressive reduction in facial erythema over the 4th month period. Compared to baseline, erythema decreased to 25% by month 2 and reached approximately 80% by month 4 (*p* < 0.01). In contrast, the control group maintained stable erythema levels throughout the study (Figure 1A).

A significant reduction in visible telangiectasias was observed in the intervention group starting from month 2, with sustained improvement through month 4 (*p* < 0.01). The control group showed no significant changes (Figure 1B).

Skin hydration increased significantly in the treated group, reaching nearly an 80% improvement from baseline by month 4 (*p* < 0.01). The control group showed a modest improvement of approximately 10%–15%, with no statistical significance (Figure 1C).

A significant improvement in skin elasticity was observed in the treatment group beginning at month 3 (*p* < 0.01), with values approaching 50% above baseline at month 4. The control group demonstrated minimal change across all time points (Figure 1D).

### 3.2 Visual Characteristics of the Skin

**FIGURE 2:**
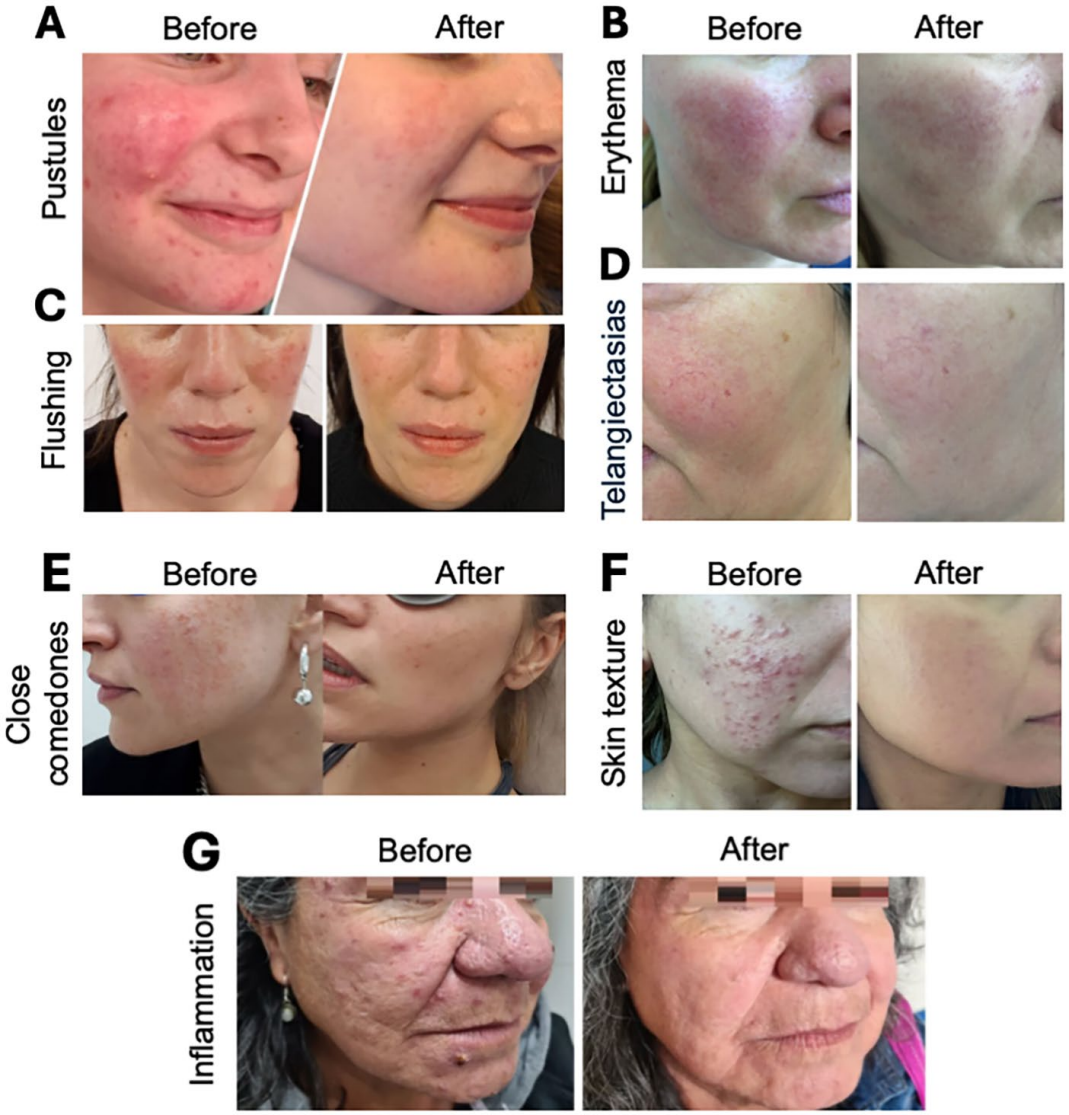
Representative clinical photographs showing improvements in rosacea signs and skin quality before and after treatment with mesotherapy with HMWNCA + SA. The parameters evaluated were pustules (A), erythema (B), flushing (C), telangiectasias (D), close comedones (E), skin texture (F) and general inflammation (G). Representative figures of our studied patients.

We found statistically significant benefits related to mesotherapy with HMWNCHA and SA. Standardized before‐and‐after images corroborate the quantitative findings, demonstrating visible reduction in erythema and telangiectasias, as well as improved skin texture. Treated individuals exhibited a noticeable decrease in inflammatory signs and enhanced skin tone uniformity (Figure 2).

### 3.3 Sensitive Manifestations of Rosacea

After 3 months, participants in the intervention group demonstrated statistically significant improvements in all self‐perceived sensitive skin symptoms. Soreness scores decreased from 7.1 ± 1.3 to 1.2 ± 0.3 (*p* < 0.05), burning from 8.3 ± 2.3 to 2.3 ± 1.2 (*p* < 0.05), itching from 7.9 ± 1.2 to 1.5 ± 0.2 (*p* < 0.05), and tingling from 6.2 ± 1.8 to 2.1 ± 0.3 (*p* < 0.05).

In the control group, a significant reduction was observed in burning and itching but not in soreness or tingling. These findings suggest that the intervention was significantly effective in alleviating multiple sensory symptoms associated with rosacea‐related sensitive skin (Table 3).

**TABLE 3:** Self‐perception numerical rating scales (NRS) after study time (3 months).

	Control before	Control after	*p*	Intervention before	Intervention after	*p*
Soreness	6.2 ± 0.6	5.8 ± 1.2	0.75	7.1 ± 1.3	1.2 ± 0.3	< 0.05
Burning	7.5 ± 1.5	4.2 ± 0.3	< 0.05	8.3 ± 2.3	2.3 ± 1.2	< 0.05
Itching	8.6 ± 0.6	3.5 ± 1.1	< 0.05	7.9 ± 1.2	1.5 ± 0.2	< 0.05
Tingling	5.4 ± 2.3	6.4 ± 3.2	0.85	6.2 ± 1.8	2.1 ± 0.3	< 0.05

*Note:* Patients rated symptoms of soreness, burning, itching, and tingling using a numerical rating scale (0–10). The intervention group showed statistically significant reduction in all symptoms after treatment (all data are presented as mean ± standard error of the mean, Wilcoxon test *p* < 0.05), while the control group only showed significant improvement in burning and itching.

### 3.4 Treatment Safety

Minor side effects were reported (Table 4) that resolved spontaneously as expectable post‐injection treatment reactions, rather than complications. No interventions or counter treatments were needed, and the incidence in 60 procedures (3 sessions in 20 patients per session) did not exceed 45%.

**TABLE 4:** Side effects report for treatment safety.

Side effect	Incidence rate (%)
Erythema	25
Edema	16.6
Injection‐site pain	45
Bruising	15
Infection	0

*Note:* Reported side effects after mesotherapy sessions in the study population *n* = 20. Incidence rate was calculated with 100 as population coefficient.

### 3.5 Vessel Diameter and Collagen Density

**FIGURE 3:**
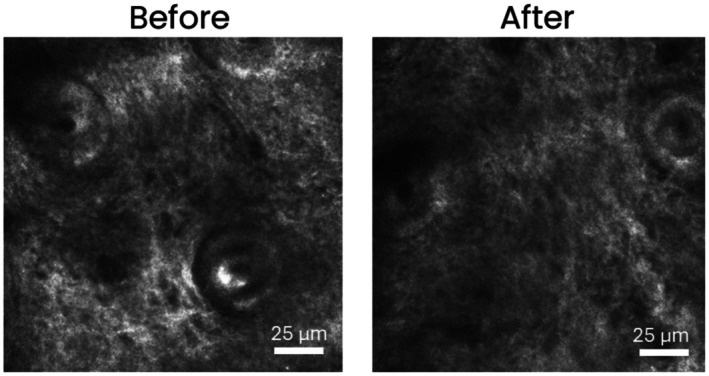
Ex vivo confocal scanning laser microscopy analysis of a skin sample before and after treatment. Image obtained using the LPE confocal laser microscope revealed structural changes and improvements in dermal architecture.

After treatment, there was a pronounced narrowing of previously dilated vessels, both small and large, including those in the dermal papillae. The overall structure of the upper dermis appeared more homogeneous, with a normal arrangement of fibers and vessels (Figure 3).

### 3.6 Cost‐Effectiveness

**FIGURE 4:**
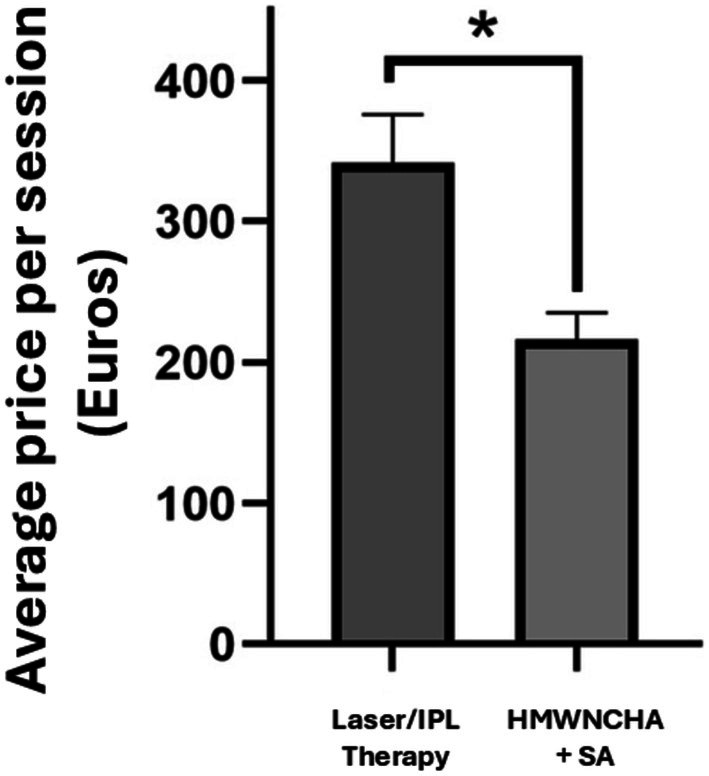
Bar graph illustrates the average cost per session (in Euros) of conventional laser or IPL therapy versus mesotherapy with HMWNCHA + SA. Data are presented as mean ± standard error of the mean. Wilcoxon test *p < 0.05.

We included a price comparison between laser therapy and mesotherapy with HMWNCHA plus SA, converted from Chilean pesos to Euros. Our results showed that the treatment offered in our study was not only superior in efficacy but also lower in cost compared to laser treatments (Figure 4).

## 4 Discussion

Our results suggest that HMWNCHA plus SA mesotherapy is a suitable adjunct treatment for rosacea in middle‐aged patients who have already tried topical treatments without a good response.

It is well accepted that external factors such as lifestyle, but also pathological conditions, can be triggers for rosacea [3, 4]. For this reason, the selection of patients for our study included multiple exclusion criteria, and more importantly, required proper adherence to the topical treatment. Our findings support the concept of personalized treatment, addressing the multiple pathogenic components of rosacea with dual topical therapy [34], while also consider the additional benefits of innovative treatments.

HMWNCHA plus SA mesotherapy improved the main features of rosacea: erythema, telangiectasia, hydration, and skin elasticity. This was the cornerstone of our intervention, as the current goals of comprehensive rosacea management are to improve these main clinical manifestations [35]. Moreover, cross‐sectional studies have reported that erythema is the most bothersome symptom with the highest impact on the patient's health burden [36].

Consistent with this, even though our study's evidence was composed mostly of quantitative measurements, we also showed that the mesotherapy intervention improved overall skin appearance. This is highly relevant, as the esthetic aspect of the disease often has the greatest psychosocial impact on a patient's quality of life [37].

One highlight of our study was the improvement in sensitive manifestations of rosacea. It has been well documented that topical treatments tend to fail in this aspect; moreover, the use of certain cosmetics can worsen these symptoms [38].

The currently established treatments for rosacea have been associated with multiple side effects such as tiredness, dizziness, lightheadedness, skin burning, pruritus, and dry skin [39, 40, 41]. Our treatment did not present severe complications, only the expected side effects of minimally invasive injectable interventions.

It has been previously reported that rosacea is characterized by large, dilated, and anfractuous capillaries [42]. Our preliminary results are in accordance with these findings, and the mesotherapy intervention appears to be capable of reversing these alterations. Despite this, our results are based on only one subject, and there is a need to increase the sample size, which was limited by the unwillingness of patients to have a biopsy taken due to scarring concerns.

In our study, the intervention group patients received the mesotherapy sessions free of charge. However, when compared to laser therapy or IPL (both highly used treatments for rosacea), our intervention was significantly less expensive than energy‐based devices. This aspect is crucial, as access to rosacea treatments in the public healthcare system is quite limited, and it is reported that the recalcitrance of rosacea to many treatment options may prompt patients to spend exorbitant amounts of money on unsubstantiated treatment regimens attempting to achieve relief [43, 44]. For us, it is crucial to propose a treatment that could have realistic applicability and be more accessible for the patients, moneywise. Nevertheless, our study does not claim our intervention is superior to laser or IPL but as a potential therapeutic alternative in cases where access to energy‐based devices is limited or patients have contraindications to their use.

Even when we provide clinical evidence of the effect of HMWNCHA plus SA on the skin, we need to further study the molecular aspects at the cellular level, as we previously established the BLT2 action mechanism in the keratinocytes [45, 46, 47].

It is important to disclose that private sector clinics in Santiago (Chile) were randomly contacted as a representative sample solely because the main researchers were in this region at the time of writing. Yet, the price of laser and mesotherapy treatments in this country may not necessarily represent the costs of the same procedures in other countries. Furthermore, differences in healthcare systems and pricing structures internationally could affect the extrapolation of our findings in other regions. Our observations require a formal cost‐effectiveness analysis to establish a solid conclusion in this matter.

Limitations of our study include the small number of participants, no independent evaluation, lack of blinding, short follow‐up, and single‐center design. Moreover, comparing our intervention with a group using laser/IPL would be highly relevant to evaluate the differences in effectiveness of adjutant therapies. Additionally, a placebo group was not included for ethical reasons, as injecting saline or doing a half‐face intervention could potentially harm or involve a negative impact on our patients.

Overall, our study opens the possibility for the use of HMWNCHA plus SA mesotherapy in the management of rosacea. However, multicenter randomized studies involving a larger number of patients, with a wider age range and phototypes, and longer follow‐up periods are needed to propose this treatment in international rosacea management guidelines.

## 5 Conclusions

HMWNCHA plus SA mesotherapy improves visual and sensory manifestations in rosacea in an adult population without comorbidities and with prior gold‐standard topical treatment without satisfactory improvement.

Moreover, it is a cost‐effective and safe treatment that can be used in skin phototypes I to III as an adjunct therapy to topical treatment based on ROSCO guidelines.

## Disclosure

The authors have nothing to report.

## Ethics Statement

Each subject signed a comprehensive written informed consent that included granting permission for the acquisition and publication of photographs. This work was conducted according to the principles stated in the Declaration of Helsinki. The study was set in a private practice setting, and an internal board of consultant physicians approved and supported the study under the code RCM56742, year 2021.

## Conflicts of Interest

A. Leguina‐Ruzzi is a Key Opinion Leader for Hyalual, MAD Skincare, Hansbiomed, and Tlab and has received stipends as a speaker unrelated to this study. A. Navarro and M. Zambrano declare no conflicts of interest.

## Data Availability

The data that supports the findings of this study are available from the corresponding author upon reasonable request.
